# Analysis of conceptual overlap among formal thought disorder rating scales in psychosis: a systematic semantic synthesis

**DOI:** 10.1038/s41537-025-00712-z

**Published:** 2025-12-15

**Authors:** Alban Voppel, Silvia Ciampelli, Tilo Kircher, Peter F. Liddle, Raffael Massuda, Frederike Stein, Sunny X. Tang, Manaan Kar Ray, Sohee Park, Lena Palaniyappan

**Affiliations:** 1 Department of Psychiatry, Douglas Mental Health University Institute, McGill University, Montreal, QC Canada; 2 Center for Clinical Neuroscience and Cognition, University of Groningen, University Medical Center Groningen, Groningen, The Netherlands; 3 Dept. of Psychiatry, Marburg University, Marburg, Germany; 4 The Institute of Mental Health, University of Nottingham, Nottingham, UK; 5 Department of Psychiatry - Federal University of Parana (UFPR) – Brazil, Curitiba, Brazil; 6 Zucker Hillside Hospital, Northwell Health, Glen Oaks, NY USA; 7 Institute of Behavioral Science, Feinstein Institutes for Medical Research, Manhasset, NY USA; 8 Department of Psychiatry, Zucker School of Medicine at Hofstra/Northwell, Hempstead, NY USA; 9 Addiction and Mental Health Services, Princess Alexandra Hospital, Metro South Hospital and Health Service, Brisbane, QLD Australia; 10 Australian Institute for Suicide Research and Prevention, Griffith University, Brisbane, QLD Australia; 11 PA-Southside Clinical Unit, Princess Alexandra Hospital, Faculty of Medicine, University of Queensland, Brisbane, QLD Australia; 12 Department of Psychology, Vanderbilt University, Nashville, TN USA; 13 Department of Psychiatry, Schulich School of Medicine & Dentistry, University of Western Ontario London, London, ON Canada; 14 Robarts Research Institute & Lawson Health Research Institute, London, ON Canada

**Keywords:** Schizophrenia, Human behaviour

## Abstract

Measuring Formal Thought Disorder (FTD), a common, cross-diagnosed symptom dimension across mental disorders, is plagued by numerous inconsistencies. Clinicians use either FTD-specific scales or items from generic scales. While these tools are based on extensive clinical observations, they suffer from inconsistent terminology. Different scales may use the same term for distinct concepts or different terms for the same concept. This lack of conceptual standardization prevents the identification of underlying FTD subconstructs. By using natural language processing, we compared the definitions, labeling and overlap of FTD symptoms, i.e., the definitions of single items, across psychopathological scales. We used a three-pronged validation approach to analyze semantic clusters of single definitions of FTD scale psychopathological items. First, we used sentence-BERT to divide 30 Thought and Language Disorder scale (TALD) items into positive or negative FTD clusters, validating this approach by checking for correspondence with published factor-analytic divisions (*approach validation*). Second, we created a sparse item-to-item similarity matrix from 103 items across seven scales to identify semantically converging cross-scale FTD items; a clinician-researcher described the resulting four clusters, and we compared our automated classification with that of six blinded experts to establish *expert-machine semantic correspondence*. Finally, we analyzed data from 98 participants (49 healthy controls and 49 schizophrenia/affective psychosis), identifying the highest-correlating Clinical Language Disorder Scale (CLANG) item for each Thought, Language and Communication (TLC) scale item and mapping these to our BERT-derived clusters to establish *data-level correspondence*. When assigning TALD items to BERT-derived positive or negative FTD groupings, we observed a 73% match with prior factor analyses. The BERT-informed clustering of cross-scale items highlighted four coherent FTD groupings: (1) muddled communication & incomprehension, (2) abrupt topic shifts, (3) inconsistent narrative structure, (4) restricted speech. Expert raters showed moderate-to-high overlap (Fleiss’ kappa = 0.617) with computational clusters. A binomial test indicated that at the level of individual participants, correlations among CLANG-TLC item pairs were significantly more likely than chance to fall into the expected semantic cluster (*p* < 0.001). FTD rating scales measure overlapping, semantically related constructs that drive item-level correlations. Semantic clustering acts as a novel method to harmonize multi-scale data and pinpoint discrepancies between expert and machine classifications. Computational linguistics has the potential to improve consistency across rating scales especially when measuring complex constructs such as FTD.

## Introduction

Formal thought disorder (FTD) is present across many mental disorders and is a hallmark symptom of schizophrenia-spectrum disorders in particular. It manifests as disruptions in the structure and flow of thought, often resulting in incoherent, fragmented or diminished speech^1^. There is no singular approach to measure the severity of FTD; clinicians use scales such as the Thought, Language, and Communication scale (TLC)^2^, the Thought and Language Disorder scale (TALD)^3^, or items from general symptom scales such as the Positive and Negative Syndrome Scale (PANSS)^4^. These scales are grounded in extensive clinical observations and historical conceptualizations of FTD^1,5–7^. Although these scales set out to capture an overlapping construct, some incorporate items that do not appear in others, provide notably different labels to the same phenomena, or describe differing phenomena using the same label. For instance, the phenomena described as “logorrhea” in TALD broadly aligns with “pressured speech” in TLC, but the differences in nosology can obscure their equivalence. These variations make it difficult to compare the scores across studies using different scales, and raises questions on the reproducibility and interpretation of empirical observations (e.g., neuroimaging correlates, outcome predictions, treatment effects) reported from various FTD scales^8^. To address this problem of ‘incommensurability’ among scales^9^, here we employ observations of FTD scales from patients, human experts as well as machines detecting patterns from human language to provide a more structured understanding of this domain.

One major source of heterogeneity among FTD scales stems from the differences in the concepts the authors set out to measure via items when constructing the rating scales. For instance, “poverty of thought” has been conceptualised as a problem in the quantity of speech (TLC), the patient’s subjective experience (TALD), or their ability to sustain a conversation (PANSS). An obvious source of conceptual divergence is the level within the thought-language-communication system that each scale has focussed on. Some authors have explicitly endorsed an aspect of thought, language or communication over the others, though the resulting scales retained the broad aim of measuring FTD. A less obvious source of divergence is the varied theoretical assumptions held by the authors of rating scales. To date, it is not clear how these conceptual differences influence the building blocks (sub-constructs) that constitute FTD. A systematic examination of the meaning conveyed by operational descriptions of items across scales can clarify this issue.

Some of the measurement heterogeneity has become evident from factor analytic approaches that focused on item validity and symptom overlap (e.g.,^1,5,10,11^ for a review see^8^). Although factor analysis can illuminate latent dimensions and guide conceptual refinements, its scope is inherently constrained by the need for substantial, overlapping datasets. To examine cross-scale consistency using this approach, the same participants need to be rated on multiple scales. This is not only time-consuming, but also prone to implicit rater bias (if done by the same rater) or unknown inter-rater differences, and is often not scalable beyond two instruments^7,12,13^.

An emerging alternative approach is the use of content analysis. Here, the substrate of analysis is the descriptive text of rating scales rather than participant-level scores. This approach has been used to explore, for example, screening questionnaires in clinical high-risk psychosis^14^, depression^15^ and neurological symptoms^16^. The advent of Natural Language Processing (NLP) approaches have advanced content analysis by enabling granular examination of semantics i.e., the meaning carried by descriptors in rating scales. Such NLP approaches employ large language models trained on extensive corpora that capture subtle semantic relationships among words and sentences, providing an objective complement to expert-driven content analysis. Models such as Bidirectional Encoder Representations from Transformers (BERT) and GPT^17,18^ offer theory-agnostic approaches for language assessment enabling concepts that drive textual descriptions to be made explicit (e.g., see^19^). By embedding each item of a rating scale in a high-dimensional semantic space, it becomes feasible to quantify item-to-item similarity in meaning (semantic clustering) across multiple rating instruments^20^. While the application of NLP to study FTD has mostly focused on patient-derived speech data^21–24^, we employ this approach to examine how different scales define, label, and describe the phenomena that constitute FTD.

Here, we apply semantic clustering to rating scales that are commonly used in operationalising the concept of FTD. Our aim is to identify the elements that constitute the “backbone” of the FTD construct across the scales. We anticipate this semantic structure to reflect the core overlapping concepts that likely influenced the itemisation of FTD construct across scales. To this end, we first demonstrate that the item-level semantic clustering approach applied to one of the most exhaustive FTD scales (TALD) identifies the relationships that define the well-established subconstructs of positive and negative FTD (*approach validation*). We then analyze 103 item-level descriptions from 7 FTD scales to identify meaningful clusters based on conceptual overlap and lack thereof (*cluster generation*). We estimate the agreement between machine-generated clusters and human-experts in assigning items to each cluster (*expert assignment*). Finally, we relate these findings to a clinical dataset in which two diverging scales - CLANG and TLC - were administered (*clinical alignment*), comparing how real-world item correlations map onto the NLP-derived clusters. To reconcile the observed differences and to aid harmonisation of FTD measurements across empirical studies, we provide the means to place individual items onto potential semantic clusters.

## Methods

### Rating scale items

We selected seven commonly used rating scales for measuring FTD: the TALD^3^, the Scale for the Assessment of Positive Symptoms and Scale for the Assessment of Negative Symptoms (SAPS-SANS)^25,26^ the PANSS^4^, the Thought and Language (TLI)^27^, the Scale for the Assessment of TLC^2^, the Assessment of Bizarre-Idiosyncratic Thinking (BIT)^28^, and the Clinical Language Disorder Rating Scale (CLANG)^29^.

For the multi-dimensional scales (SAPS-SANS and PANSS), only subscales directly related to disorganized thinking or FTD were included. In total, we extracted 103 FTD-related items from these scales (see Supplemental Table 1 for a list of included items). Since these scales vary in their level of detail—some providing extensive examples, others using concise descriptors—we removed illustrative parenthetical examples to maintain consistency in focus and length. For instance, when processing the TALD item “restricted thinking”, we removed the words “(e.g., a depressive patient who is preoccupied with his indigestion)”. This allowed us to retain only the core phrasing of each item for semantic comparison.

### Semantic embeddings

All item-level descriptions were converted to lowercase for consistency. We selected a sentence-level BERT model, specifically the *all-mpnet-base-v2* model (https://huggingface.co/sentence-transformers/all-mpnet-base-v2), because the symptom descriptions predominantly comprise single sentences or short text segments, making a sentence-centric approach particularly suitable. The *all-mpnet-base-v2* pre-trained model maps text (at the sentence or paragraph level) to a 768-dimensional dense vector space and was trained on a broad range of natural language tasks, thereby capturing contextual and semantic nuances within and between sentences^17^. Consequently, each FTD item description was transformed into a unique 768-dimensional embedding. By using a single, standardized embedding approach, we avoid biases that might arise from employing multiple specialized models or custom vocabularies.

### Approach validation - positive and negative FTD

We assessed sentence cosine similarity between 30 TALD item descriptions and published descriptions of the widely used constructs of positive and negative FTD^3^. We employed the TALD alongside factor descriptions ensuring consistency as originally described by the same authors. Specifically, we compared each TALD item-level description to the following text for positive FTD—*“Positive FTD is best represented by derailment and loosening of associations, an increased amount of produced speech (e.g., logorrhoea, pressured speech), the use of new words (neologisms), and stilted speech phenomena (manneristic speech)”*—and for negative FTD—*“Negative FTD has been conceptualised as a quantitative deficit in speech and thought production (e.g., poverty of speech, slowed thinking, and blocking)”*^30^. Each of the 30 TALD items was then assigned to a positive or negative FTD group, according to whichever similarity score (positive or negative) was higher. The resulting two-way classification was subsequently compared with a factor analysis–derived grouping of the same items^3^.

### Clustering across scales

Using the 768-dimensional embeddings, we computed pairwise semantic similarity for all 103 items. Similarity was defined as the cosine angle between vectors, normalized to yield similarity scores ranging from 0 (no similarity) to 1 (identical vectors). This resulted in a 103 × 103 connectivity matrix whose cells indicated how semantically similar each item was to every other item.

To facilitate interpretation, we sparsified this matrix, preserving only the strongest semantic connections. Specifically, we treated the TLC items as a backbone, ensuring that each item from the other six scales retained only its single highest similarity link to an item in TLC. This procedure effectively filtered out less relevant connections, aiding in the discovery of cohesive clusters (or “communities”) of related items. The TLC was chosen as backbone for its widespread use and comparable number of items to other rating scales, as well as the availability of the patient dataset with CLANG & TLC.

### Expert-machine semantic grouping

The resulting clusters using the TLC backbone were described by author LP, avoiding any words used in item titles. We then used expert raters, members of the DISCOURSE consortium clinical harmonization group to assign all 103 items to one of the four backbone groups based on this description, allowing us to compare BERT group structure to that of human raters, by looking at overlapping group membership of machine and human-picked clusters.

### Clinical alignment and clustering

In a separate sample of 98 participants (49 healthy controls and 49 individuals with schizophrenia or affective psychosis), both CLANG and TLC scales were administered. Participants were recruited from community-based clinics and hospitals through the Oxford Mental Health Services in Oxford, United Kingdom as part of the Cerebral Asymmetry and Functional Language in Psychosis (CAFLIP) study and gave written informed consent. For each TLC item, we computed its correlation with all CLANG items across the participant group and identified the strongest CLANG correlate, pairing items across the scales. We then checked whether each TLC–CLANG pair appeared in the same BERT-derived cluster, providing a real-world assessment of how clinical item relationships corresponded to the NLP-based semantic groupings.

## Results

### Rating scales items

We extracted and embedded 103 items from the seven FTD rating scales (TALD, SAPS-SANS, PANSS, TLI, TLC, BIT, and CLANG) and observed substantial variation in their descriptive richness. CLANG items were the most concise (14.4 words on average), while TLC items were the longest (95.1 words on average, up to 242 words). SAPS-SANS showed similarly high word counts (90.2 on average - note that the TLC & SAPS-SANS share the same author), with BIT, TALD, PANSS, and TLI occupying middle ranges. This broad range in word count and exemplification underscores how each scale captures FTD with different levels of detail and granularity.

### Semantic embeddings

**Fig. 1:**
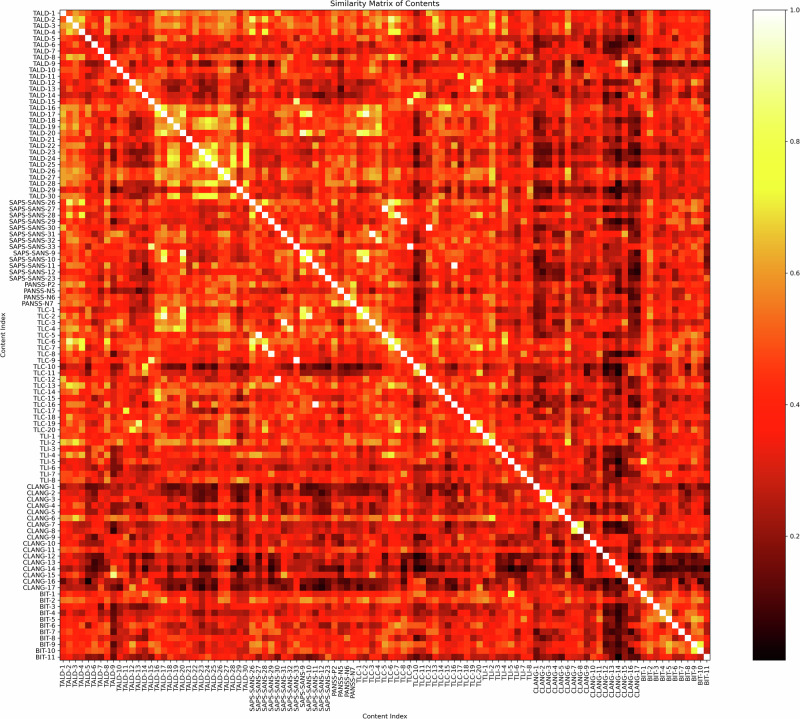
Similarity matrix of individual FTD rating items. The matrix includes 103 FTD items, with cosine similarity values ranging from 0 (very dissimilar) to 1 (very similar). Several item pairs show near-identical similarity (notably SAPS-SANS and TLC items), indicated by values at or close to 1. Yellow clusters along the diagonal highlight high within-scale coherence (e.g., TALD items 16 to 30), whereas dark vertical and horizontal bands illustrate greater semantic divergence (e.g., CLANG items). TALD thought and language disorder, SAPS-SANS scale for the assessment of positive symptoms and scale for the assessment of negative symptoms, PANSS positive and negative syndrome scale, TLC thought, language, and communication, TLI thought, language, and communication disorder index, BIT assessment of bizarre-idiosyncratic thinking, CLANG clinical language disorder rating scale.

**Fig. 2:**
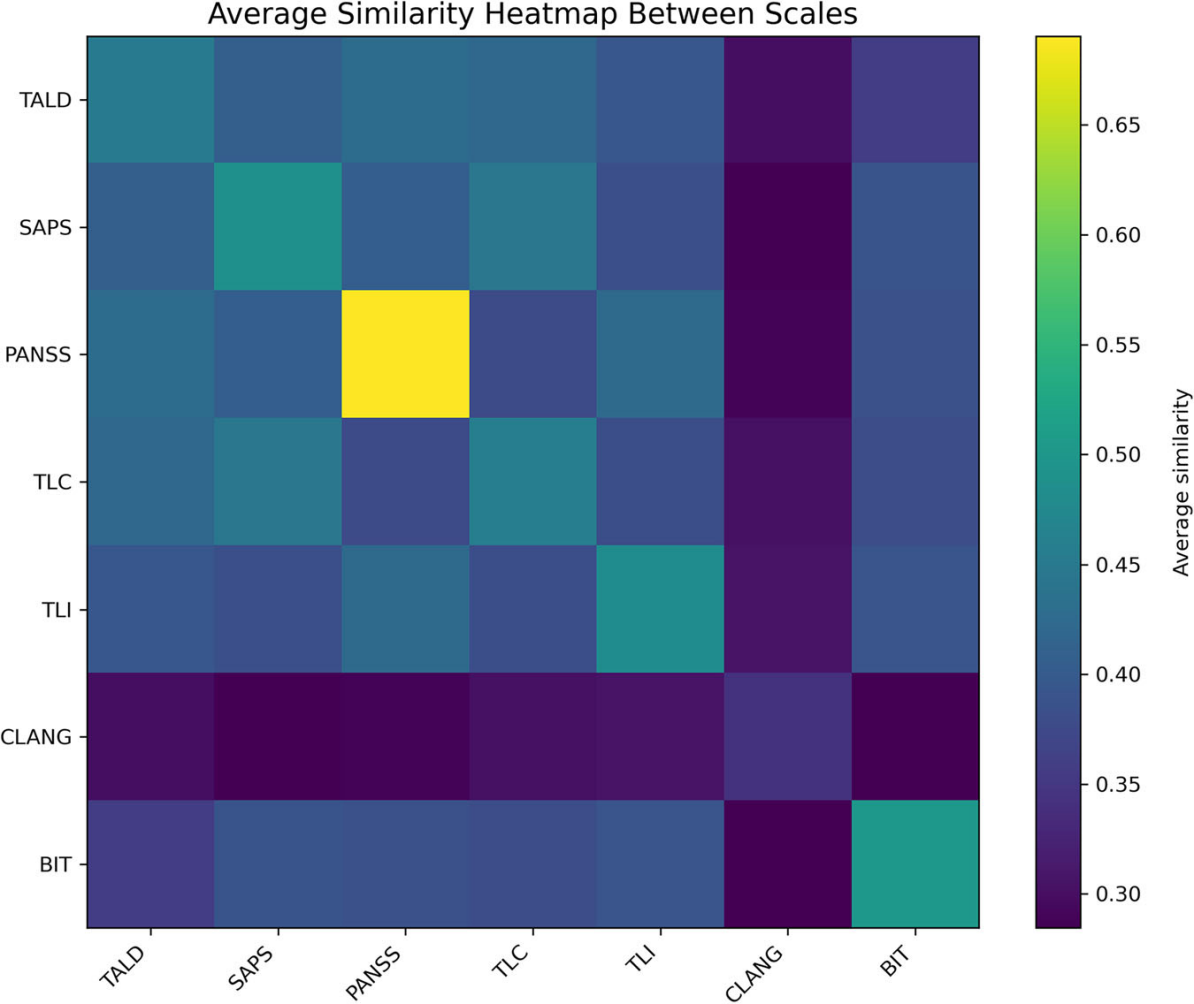
Scale-level similarity matrix of FTD rating scales. Comparing the average embeddings for each of the seven FTD rating scales. Diagonal cells indicate the average of the scale’s within-scale item semantic consistency and are thus not 1; off-diagonal cells highlight the degree of semantic overlap between different scales. Warmer colors (closer to 1) signify greater semantic similarity between scales, whereas cooler colors (closer to 0) indicate more divergent conceptual semantic coverage. TALD thought and language disorder, SAPS-SANS scale for the assessment of positive symptoms and scale for the assessment of negative symptoms, PANSS positive and negative syndrome scale, TLC thought, language, and communication, TLI thought, language, and communication disorder index, BIT assessment of bizarre-idiosyncratic thinking, CLANG clinical language disorder rating scale.

Item-level descriptions from 7 scales were embedded in 768-dimensional vector space, and the resulting cosine similarity scores grouped in a matrix illustrating the relationships among individual items (Fig. 1). Higher similarity values (closer to 1) indicate stronger semantic overlap, revealing potential clusters of conceptually related symptoms across the different scales. To examine broader patterns at the scale level, we computed a second similarity matrix by first averaging item embeddings within each rating scale to form a scale “centroid”, and then calculating cosine similarity between every pair of centroids (Fig. 2). The diagonal entries in this matrix reflect the mean semantic consistency of the items within each scale, i.e., semantic distance between TLI item 1 to TLI items 2–7, repeated for each item within the scale. The off-diagonal cells indicate the degree of overlap of “centroids” between different scales.

### Approach validation - positive and negative FTD

When classifying TALD items into positive or negative FTD groups based on higher cosine similarity, 23 of 30 items (73.3%) matched the factor analysis–derived classification reported by Kircher et al. (2014), representing a statistically significant association (*χ*²(1, *N* = 30) = 6.04, *p* = 0.014). For instance, item TALD-14 (“Neologisms”) had a higher similarity to the positive FTD text (0.367) than to the negative FTD text (0.220), consistent with the factor-analytic assignment. This significant overlap serves as a proof-of-concept that semantic similarity–based categorizations can align closely with traditional factor-analytic divisions of FTD.

### Clustering across scales

**Fig. 3:**
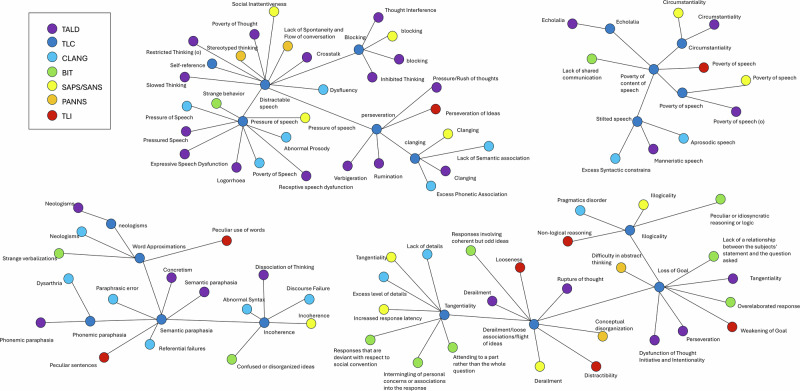
Semantic network of all items to TLC backbone. The network obtained by linking each item from the seven FTD scales to its single most similar TLC item, revealing four distinct clusters of semantically related symptoms. In the bottom-left cluster (Group 1), items reflect muddled communication with incomprehensible elements, meaning distortion, and word substitution that heighten confusion. The middle-top cluster (Group 2) highlights abrupt topic shifts, repeated words or subjects, speech interruptions, and lapses in recalling previous statements. The top-right cluster (Group 3) centers on unfounded conclusions, irrelevant replies, lack of thought continuity, and disjointed ideas, resulting in inconsistent narrative structure. Finally, the bottom-right cluster (Group 4) illustrates off-topic wandering, weak context association, and minimal connecting “cementing words,” exacerbating difficulties in word sequencing. Items are color-coded by the rating scale prefix (e.g., TALD, TLC, CLANG, BIT, SAPS-SANS, PANSS, TLI), illustrating how conceptually similar symptoms from different scales co-locate in the same region of the network. For a full description of these four groups and the corresponding items, see Table 1. For a version of this division where edges represent similarity, see supplemental Fig. 1.

The result of our network sparsification, using TLC as a backbone and linking each item from the seven scales only to its single highest-similarity TLC item (Fig. 3), significantly reduced the complexity of the full similarity matrix. This streamlined network revealed four internally connected clusters of item-level symptoms, reflecting coherent groupings of semantically related FTD features across different rating scales. See Table 1 for a list of individual items for each of the four groups.

**Table 1:** Four clusters based on semantic embeddings with TLC as backbone.

Description	Scale index	Rating-scale items according to TLC-clustering
*Group 1* features result in muddled communication with some incomprehensible elements. Meaning distortion, word substitution, and weak context association occur. The absence of crucial “cementing words” worsens confusion, causing difficulties in word sequencing.	TLI-6	Peculiar sentences
	TLI-5	Peculiar use of words
	TLC-7	Incoherence (Word salad, Jargon Aphasia)
	TLC-20	Semantic paraphasia
	TLC-19	Phonemic paraphasia
	TLC-11	Word approximations (Paraphasia, Metonyms)
	TLC-10	Neologisms
	TALD-4	Dissociation of thinking (Incoherence/Distraction)
	TALD-21	Concretism
	TALD-14	Neologisms
	TALD-13	Phonemic paraphasia
	TALD-12	Semantic paraphasia
	SAPS-SANS-28	Incoherence
	CLANG-6	Discourse failure (Loss of schematic organization)
	CLANG-5	Referential failures
	CLANG-2	Abnormal syntax
	CLANG-17	Paraphasic error
	CLANG-16	Neologisms
	CLANG-13	Dysarthria
	BIT-6	Confused or disorganized ideas.
	BIT-1	Strange verbalizations
*Group 2* features involve abrupt topic shifts in response to stimuli, repeated words or subjects, speech interruptions, and memory lapses regarding previous speech.	TLI-3	Perseveration of ideas
	TLC-9	Clanging
	TLC-4	Distractible speech
	TLC-3	Pressure of speech
	TLC-18	Self-reference
	TLC-16	Blocking
	TLC-14	Perseveration
	TALD-9	Pressured speech
	TALD-7	Verbigeration
	TALD-5	Crosstalk
	TALD-30	Pressure/Rush of thoughts
	TALD-29	Thought interference
	TALD-27	Expressive speech dysfunction
	TALD-26	Receptive speech dysfunction
	TALD-25	Inhibited thinking
	TALD-24	Poverty of thought
	TALD-23	Rumination
	TALD-22	Blocking
	TALD-19	Slowed thinking
	TALD-18	Restricted thinking (o)
	TALD-15	Clanging
	TALD-10	Logorrhoea
	SAPS-SANS-33	Clanging
	SAPS-SANS-32	Distractible speech
	SAPS-SANS-31	Pressure of speech
	SAPS-SANS-23	Social inattentiveness
	SAPS-SANS-11	Blocking
	PANSS-N7	Stereotyped thinking
	PANSS-N6	lack of spontaneity and flow of conversation
	CLANG-4	Lack of semantic association
	CLANG-15	Pressure of speech
	CLANG-14	Poverty of speech
	CLANG-12	Dysfluency
	CLANG-10	Abnormal prosody
	CLANG-1	Excess phonetic association
	BIT-11	Strange behaviour
*Group 3* features include unfounded conclusions, irrelevant replies, a lack of thought continuity, off-topic wandering, disjointed ideas, and a dearth of meaningful connections, resulting in inconsistent narrative structure.	TLI-7	Non-logical reasoning (peculiar logic)
	TLI-4	Looseness
	TLI-2	Weakening of goal
	TLC-8	Illogicality
	TLC-6	Derailment/Loose association/Flight of ideas
	TLC-5	Tangentiality
	TLC-13	Loss of goal
	TALD-8	Rupture of thought
	TALD-6	Perseveration
	TALD-3	Tangentiality
	TALD-28	Dysfunction of thought initiative and Intentionality
	TALD-2	Derailment
	SAPS-SANS-29	Illogicality
	SAPS-SANS-27	Tangentiality
	SAPS-SANS-26	Derailment
	SAPS-SANS-12	Increased response latency
	PANSS-P2	Conceptual disorganization
	PANSS-N5	Difficulty in abstract thinking
	CLANG-8	Lack of details (in given context)
	CLANG-7	Excess level details (in given context)
	CLANG-11	Pragmatics disorder
	BIT-9	Attending to a part of rather than the whole question
	BIT-8	Intermingling of personal concerns/associations into the response.
	BIT-7	Overelaborated response.
	BIT-5	Peculiar or idiosyncratic reasoning or logic.
	BIT-4	Responses that are deviant with respect to social convention.
	BIT-3	Responses involving coherent but odd ideas
	BIT-10	Lack of a relationship between subject’s statement and question asked
*Group 4* features entail restricted speech, brief concrete answers, omission of additional information, overly concise replies, uninformative or indirect responses with vague, repetitive, or abstract language, hindering meaningful information extraction by interviewers.	TLI-1	Poverty of speech
	TLC-2	Poverty of content of Speech
	TLC-17	Stilted speech
	TLC-15	Echolalia
	TLC-12	Circumstantiality
	TLC-1	Poverty of speech
	TALD-20	Poverty of speech (o)
	TALD-17	Poverty of content of speech
	TALD-16	Echolalia
	TALD-11	Manneristic speech
	TALD-1	Circumstantiality
	SAPS-SANS-9	Poverty of speech
	SAPS-SANS-30	Circumstantiality
	SAPS-SANS-10	Poverty of speech content
	CLANG-9	Aprosodic speech
	CLANG-3	Excess syntactic constraints (Excess grammar)
	BIT-2	Lack of shared communication.

Descriptions of clusters were generated by LP and used for clinical rater agreement; for a visual representation of clusters, see Fig. 3 and supplemental Fig. 1.

### Expert-machine semantic grouping

Following the identification of the four BERT-derived clusters based on TLC embeddings, one author (LP) provided short, non-scale-specific descriptions for each group, in brief (1) muddled communication & incomprehension. (2) Abrupt topic shifts. (3) Inconsistent narrative structure, (4) restricted speech (see Table 1 for full descriptions). These descriptions were generated through a visual inspection of the clusters, without accessing the full list of items that belonged to each cluster. The descriptions were constructed in a manner that avoided the verbatim repetition of item names. Functional effects (e.g., difficulties in sequencing) rather than cognitive or mechanistic processes (e.g., lack of associations) were included in the descriptions. Six expert raters from the DISCOURSE consortium Clinical Harmonization Group then independently assigned each of the 103 items to one of these four clusters. Their assignments were compared with the original BERT-based groupings; Table 2 summarizes both overall and cluster-specific Fleiss’ kappa values. Raters demonstrated substantial consensus (*κ* = 0.617, 95% CI: 0.585–0.648 for all 103), while agreement per cluster ranged from 0.476 to 0.716, suggesting some categories may be more intuitively distinguished than others. Top 3 (best total agreement between raters & BERT cluster assignment) were Poverty of Speech (TLC-1), Neologisms (TALD-14) and Tangentiality (TLC-5) while 3 most disagreed items (worst agreement between raters & BERT cluster assignment) were Distractibility (TLI-8), Lack of details (CLANG-8) and PANSS-N7 (Stereotyped Thinking)

**Table 2:** Human-BERT group assignment agreement.

Ratings	Fleiss' kappa	SE	95% CI	
			Lower	Upper
Overall	0.617	0.016	0.585	0.648
Group 1	0.636	0.028	0.582	0.690
Group 2	0.476	0.028	0.422	0.530
Group 3	0.589	0.028	0.535	0.643
Group 4	0.716	0.028	0.662	0.770

Overall and per-group Fleiss’ kappa values on agreement between six human raters and the BERT-based 4-group cluster assignments for 103 FTD items. Confidence intervals (CI) are asymptotic and indicate the precision of each kappa estimate.

### Clinical alignment and clustering

**Fig. 4:**
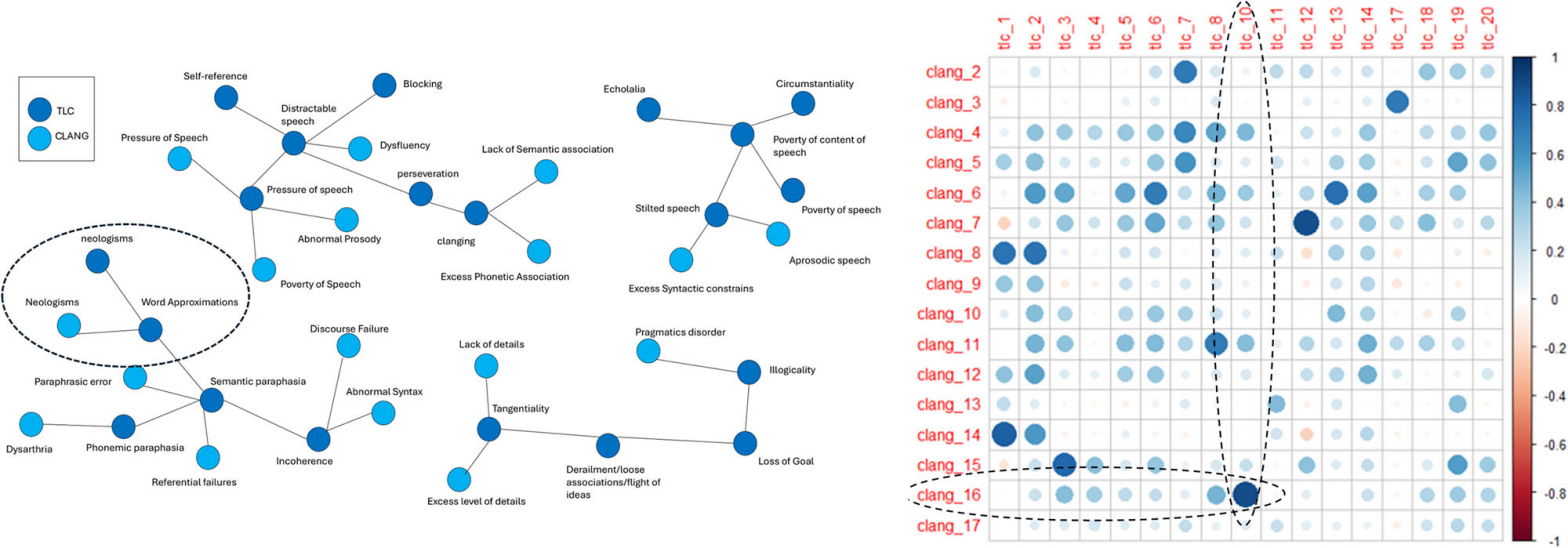
TLC-CLANG semantic cluster and correlations. Left; clustering based on semantic distance rating, using the TLC as a backbone. Circled area highlights neologism symptoms grouped in one cluster. Right, individual clinical item correlations between CLANG and TLC in patient & control sample (*n* = 98). TLC-10 (Neologisms) and CLANG-16 (Neologisms) circled. Note that Clang-1 (phonetic association), TLC-9 (clanging), TLC 15 (echolalia) and TLC 16 (Blocking) were excluded from the correlational analysis due to the absence of any cases having a non-zero score.

In the sample of 98 participants (49 healthy controls and 49 individuals with Schizophrenia, Bipolar Disorder or Depression; see Table 3 for demographics), both CLANG and TLC items were rated. Four items -CLANG-1 (phonetic association), TLC-9 (clanging), TLC-15 (echolalia), and TLC-16 (blocking)- were excluded from further analysis because no participant received a non-zero score on these items. For the remaining items, each individual rating item was correlated with each other item. The highest CLANG-TLC item correlations were identified and compared to the four-domain semantic clustering applied to the same scales, see Fig. 4. Eleven out of 17 correlational item pairs fell into the same semantic domain, a result that significantly exceeded the 25% chance-level expectation (binomial test, *p* < 0.001).

**Table 3:** CLANG-TLC participant demographic and clinical characteristics.

Group	Patients	Healthy controls
*N*	49	49
Sex (M:F)	33:16	27:20*
Age in years (Mean, SD)	33.63 (8.32)	31.02 (9.43)*
Diagnosis	Schizophrenia = 27 Bipolar disorder = 18 Depression = 4	
TLC total score mean, (SD)	6.47 (6.94)	4.51 (5.11)
Total CLANG score (Mean, SD)	6.65 (5.15)	4.00 (4.73)

Sample from the CAFLIP study.

*CLANG* clinical language disorder rating scale, *TLC* thought, language, and communication, *SD* standard deviation.

*Two healthy controls had missing data for sex and age.

## Discussion

In this study, we compared items of symptom scales based on their semantic content rather than on participant scores, leveraging sentence-level BERT embeddings of FTD rating scales in different ways. First, we effectively distinguished the well-known positive and negative dimensions of FTD within the TALD scale, aligning with previous factor-analytic findings, thus providing a proof-of-concept for the semantic embedding approach. We then applied the same approach across seven rating scales, using TLC items as a backbone to sparsify the similarity matrix and reveal four distinct symptom clusters (Fig. 3), reducing the complexity of cross-scale comparisons. When evaluated against expert human judgment, these NLP-derived clusters showed substantial alignment with ratings, indicating that semantic similarity–based groupings largely matched expert perceptions. Finally, using a separate dataset with both CLANG and TLC, we found that items with the highest cross-scale correlations generally fell into the same BERT-derived cluster, suggesting meaningful convergence between real-world clinical data and our data-driven semantic domains.

In doing so, our study addresses four major points. First, NLP-based approaches offer an integrative, theory-agnostic framework for comparing conceptually related content across diverse rating instruments. Second, the identification of four distinctive semantic clusters, each containing items from different scales, shows that seemingly heterogeneous rating tools often capture overlapping constructs; for instance, “derailment” may be called “loose associations” or “flight of ideas,” yet here they converge within a single NLP-derived cluster. Third, our alignment with expert consensus and published factors suggests that clinicians’ intuitive groupings and conventional psychometric divisions do correspond, to a substantial degree, with computationally derived similarity networks—strengthening the claim that these clusters hold practical meaning. Fourth, analyzing real-world clinical data (the CLANG-TLC correlation set) revealed that items with the strongest cross-scale associations largely fell into the same NLP-derived cluster, reinforcing that these semantic groupings reflect meaningful patterns observable in patient populations.

While we provide a novel cluster and group division, it is not our intent to claim that we now know what FTD is, or how it should be definitely divided, factored, or conceptualized. Rather, our approach illustrates how NLP can serve as an “intuition pump” or tool for thinking^31^, guiding the integration of diverse clinical views, factor analyzes and historical frameworks towards a data-driven model of FTD.

Semantic analysis can compare and align items across different scales when they were not administered to the same participants, while factor analysis and latent profile analysis require a single, multivariate dataset and typically large samples. Practically, we see semantic analysis as a mapping tool to generate and refine groupings and correspondence between rating scale items. When joint datasets are available, those hypotheses should be confirmed with factor analysis. Interestingly and partially self-referential, the linguistic patterns captured in LLMs that allow them to quantify semantic distance are a subset of the patterns described as being breached in the description of FTD symptoms. Our approach adds to the toolbox for clarifying the clinical conceptualization of disordered thought and provides a means to derive ‘concept clusters’ of items across scales. Just as prior studies have examined short conceptual phrases to identify core semantic themes^19^, our approach demonstrates how large language models can distill rating-scale descriptions into interpretable clusters; a step forward in characterizing the varied symptom measurement tools for psychiatric disorders.

### Strengths and weaknesses

One strength of this work lies in its clear demonstration that items from seven different FTD rating scales can be reconciled semantically despite disparate clinical histories and descriptive styles. The inclusion of expert rater groupings and TALD-based positive–negative classifications bolsters the credibility of the NLP-based approach. Our findings also provide a springboard for practical scale development: reviewing item clusters may help condense multiple rating instruments into a smaller, more cohesive item set.

However, a few limitations warrant mention. First, antonymous statements (e.g., “responses are too slow” vs. “responses are too fast”) can appear highly similar to embedding models - in these example snippets, the cosine similarity of 0.931 using the all-mpnet-base-v2 model illustrates how purely text-based approaches may cluster together opposite ends of a scale that could be conceptualized as separate in a positive-negative FTD division. Second, the item-level focus excluded severity descriptors, which contain additional semantic information regarding symptoms. Consider that the description for the most severe rating of TALD item 4, “Dissociation of Thinking” reads: *“Scattered speech: Syntax is absent (paragrammatism, parasyntax), resulting in an incomprehensible, meaningless word and syllable mixture (“word salad”)*^3^ and includes terms and words not included in our approach where we take only the base item description, but which would likely lead to higher cosine similarity with other rating scale item descriptions containing syntactic terms. Third, our clinician-described, embedding-seeded clusters warrant caution. Group 4, for example (described as “restricted/poverty of speech”), reflects reduced quantity or diminished meaning despite intact production and can overlap with Group 1 (“muddled communication”). As with any division and description of a complex, overlapping phenomenon like FTD, overlap and alternate interpretations of clusters remain present. Finally, some of the scales were conceived in different languages before translation to English, and all scales were composed by writers and researchers with different literary and scientific styles. Despite these challenges, the measured convergence between computational and human-driven groupings is promising.

### Future directions

This item-level unification framework can be extended in several important ways. Semantic embeddings have been used to characterize clinical speech directly^32^; the approach taken here aimed to tackle an upstream problem, semantic analysis of the rating scales used to characterize these clinical samples. However, our approach could be complementary, i.e., by measuring distance between patient speech and rating clusters. Extending the CLANG-TLC approach with more patient-level data will clarify whether semantically similar items show correlated patient ratings across all rating scales. It may be equally useful to explore whether specific clusters map onto distinct neural substrates (e.g., such as connectivity within language-relevant networks), offering a biological anchor for these conceptual groupings^9,33^ In addition, identifying items with low connectivity could highlight underdefined or infrequent symptom domains that warrant refinement, integration or outright removal, thus reducing taxonomic incommensurability^9^ and refining a conceptual “hub” with core aspects of disorganized thinking. Taken together, these steps would not only refine item-level semantics but could also guide the design of more consistent, multidimensional assessment of FTD.

### Conclusion

By applying sentence-level embeddings to unify different FTD rating scales, we offer a novel conceptual framework to investigate disorganized thinking. Mapping 103 items from seven scales into four overarching domains reveals a shared semantic space that converges with expert consensus, while the positive–negative division correlates with prior factor-analytic findings. In addition, the alignment of CLANG-TLC correlations with these domains affirms the real-world applicability of our NLP-based clusters. This approach aims to enrich our theoretical grasp of how different theoretical and clinical perspectives on FTD intersect. Going forward, these insights derived from semantic embedding may serve as a flexible scaffold to refine how FTD is classified, discussed, and examined experimentally—expanding our tools for disentangling this complex yet foundational symptom domain in mental disorders.

## Supplementary information

Supplemental materials

## Data Availability

Inquiries regarding anonymous CAFLIP data access should be directed to Lena Palaniyappan - lena.palaniyappan@mcgill.ca.

## References

[1] Kircher, T., Bröhl, H., Meier, F. & Engelen, J. Formal thought disorders: from phenomenology to neurobiology. *Lancet Psychiatry* **5**, 515–526 (2018). 29678679 10.1016/S2215-0366(18)30059-2

[2] Andreasen, N. C. Scale for the assessment of thought, language, and communication (TLC). *Schizophr. Bull.* **12**, 473–482 (1986). 3764363 10.1093/schbul/12.3.473

[3] Kircher, T. et al. A rating scale for the assessment of objective and subjective formal thought and language disorder (TALD). *Schizophr. Res.* **160**, 216–221 (2014). 25458572 10.1016/j.schres.2014.10.024

[4] Kay, S. R., Fiszbein, A. & Opler, L. A. The positive and negative syndrome scale (PANSS) for schizophrenia. *Schizophr. Bull.* **13**, 261–276 (1987). 3616518 10.1093/schbul/13.2.261

[5] Covington, M. A. et al. Schizophrenia and the structure of language: the linguist’s view. *Schizophr. Res.* **77**, 85–98 (2005). 16005388 10.1016/j.schres.2005.01.016

[6] Docherty, N. M. Cognitive impairments and disordered speech in schizophrenia: thought disorder, disorganization, and communication failure perspectives. *J. Abnorm. Psychol.* **114**, 269–278 (2005). 15869357 10.1037/0021-843X.114.2.269

[7] Rodriguez-Ferrera, S., McCarthy, R. A. & McKenna, P. J. Language in schizophrenia and its relationship to formal thought disorder. *Psychol. Med.* **31**, 197–205 (2001). 11232908 10.1017/s003329170100321x

[8] Zamperoni, G., Tan, E. J., Rossell, S. L., Meyer, D. & Sumner, P. J. Evidence for the factor structure of formal thought disorder: a systematic review. *Schizophr. Res.* **264**, 424–434 (2024). 38244319 10.1016/j.schres.2024.01.006

[9] Wulff, D. U. & Mata, R. Semantic embeddings reveal and address taxonomic incommensurability in psychological measurement. *Nat. Hum. Behav*. 1–11 10.1038/s41562-024-02089-y (2025). 10.1038/s41562-024-02089-y PMC12106064 40069366

[10] Andreasen, N. C. Thought, language, and communication disorders: II Diagnostic significance. *Arch. Gen. Psychiatry* **36**, 1325–1330 (1979). 496552 10.1001/archpsyc.1979.01780120055007

[11] Peralta, V. & Cuesta, M. J. Negative symptoms in schizophrenia: a confirmatory factor analysis of competing models. *Am. J. Psychiatry* **152**, 1450–1457 (1995). 7573583 10.1176/ajp.152.10.1450

[12] Peralta, V., Cuesta, M. J. & de Leon, J. Formal thought disorder in schizophrenia: a factor analytic study. *Compr. Psychiatry* **33**, 105–110 (1992). 1544294 10.1016/0010-440x(92)90005-b

[13] Roche, E. et al. The factor structure and clinical utility of formal thought disorder in first episode psychosis. *Schizophr. Res.* **168**, 92–98 (2015). 26260080 10.1016/j.schres.2015.07.049

[14] Bernardin, F., Gauld, C., Martin, V. P., Laprévote, V. & Dondé, C. The 68 symptoms of the clinical high risk for psychosis: low similarity among fourteen screening questionnaires. *Psychiatry Res.* **330**, 115592 (2023). 37948888 10.1016/j.psychres.2023.115592

[15] Fried, E. I. The 52 symptoms of major depression: Lack of content overlap among seven common depression scales. *J. Affect. Disord.* **208**, 191–197 (2017). 27792962 10.1016/j.jad.2016.10.019

[16] Chrobak, A. A., Krupa, A., Dudek, D. & Siwek, M. How soft are neurological soft signs? Content overlap analysis of 71 symptoms among seven most commonly used neurological soft signs scales. *J. Psychiatr. Res.* **138**, 404–412 (2021). 33962127 10.1016/j.jpsychires.2021.04.020

[17] Devlin, J., Chang, M. W., Lee, K. & Toutanova, K. BERT: pre-training of deep bidirectional transformers for language understanding. In *NAACL HLT 2019 - 2019 Conference of the North American Chapter of the Association for Computational Linguistics: Human Language Technologies* Vol. 1, 4171–4186 (2019).

[18] Floridi, L. & Chiriatti, M. GPT-3: its nature, scope, limits, and consequences. *Minds Mach.* **30**, 681–694 (2020).

[19] Bolt, T. & Uddin, L. Q. “The brain is…”: a survey of the brain’s many definitions. *Neuroinformatics* **23**, 4 (2025). 39798046 10.1007/s12021-024-09699-x PMC11724787

[20] Böke, A. et al. Enhancing diagnostic precision: using large-language models to evaluate content overlap in mental health questionnaires. *JMIR* https://preprints.jmir.org/preprint/79868 (2025). 10.2196/79868 PMC12697914 41380022

[21] Bedi, G. et al. A window into the intoxicated mind? Speech as an index of psychoactive drug effects. *Neuropsychopharmacology* **39**, 2340–2348 (2014). 24694926 10.1038/npp.2014.80 PMC4138742

[22] Corcoran, C. M. & Cecchi, G. A. Using language processing and speech analysis for the identification of psychosis and other disorders. *Biol. Psychiatry Cogn. Neurosci. Neuroimaging* **5**, 770–779 (2020). 32771179 10.1016/j.bpsc.2020.06.004 PMC7430500

[23] Corona Hernández, H. et al. Natural language processing markers for psychosis and other psychiatric disorders: emerging themes and research agenda from a cross-linguistic workshop. *Schizophr. Bull.* **49**, S86–S92 (2023). 36946526 10.1093/schbul/sbac215 PMC10031727

[24] Voppel, A. E., de Boer, J., Brederoo, S., Schnack, H. & Sommer, I. Quantified language connectedness in schizophrenia-spectrum disorders. *Psychiatry Res*. **304**, 114130 (2021). 10.1016/j.psychres.2021.114130 34332431

[25] Andreasen, N. C. *Scale for the Assessment of Positive Symptoms (SAPS)*. 10.1037/t48377-000 (1984).

[26] Andreasen, N. C. *Scale for the Assessment of Negative Symptoms (SANS)*. 49–58 (1984). 2695141

[27] Liddle, P. F. et al. Thought and Language Index: an instrument for assessing thought and language in schizophrenia. *Br. J. Psychiatry* **181**, 326–330 (2002). 12356660 10.1192/bjp.181.4.326

[28] Marengo, J. T., Harrow, M., Lanin-Kettering, I. & Wilson, A. Evaluating bizarre-idiosyncratic thinking: a comprehensive index of positive thought disorder. *Schizophr. Bull.* **12**, 497–511 (1986). 3764365 10.1093/schbul/12.3.497

[29] Chen, E. Y. H. et al. Language disorganisation in schizophrenia: validation and assessment with a new clinical rating instrument. *Hong Kong J. Psychiatry* **6**, 4–13 (1996).

[30] Kircher, T., Stein, F. & Nagels, A. Differences in single positive formal thought disorder symptoms between closely matched acute patients with schizophrenia and mania. *Eur. Arch. Psychiatry Clin. Neurosci.* **272**, 395–401 (2022). 33961098 10.1007/s00406-021-01263-x PMC8938354

[31] Dennett, D. C. *Intuition Pumps And Other Tools for Thinking* (W. W. Norton & Company, 2013).

[32] Palominos, C. et al. Approximating the semantic space: word embedding techniques in psychiatric speech analysis. *Schizophrenia* **10**, 114 (2024). 39622800 10.1038/s41537-024-00524-7 PMC11612388

[33] Stein, F. et al. Transdiagnostic types of formal thought disorder and their association with gray matter brain structure: a model-based cluster analytic approach. *Mol. Psychiatry* **30**, 4286–4295 (2025). 40210978 10.1038/s41380-025-03009-w PMC12339403

