# Supplementary material for: Analysis of conceptual overlap among formal thought disorder rating scales in psychosis: a systematic semantic synthesis
Source: Schizophrenia (Heidelb). 2025 Dec 15;12(1):9. doi: 10.1038/s41537-025-00712-z (PMC12820080; doi:10.1038/s41537-025-00712-z)
Supplement: Supplementary file 1 — Supplemental materials [file 41537_2025_712_MOESM1_ESM.doc]

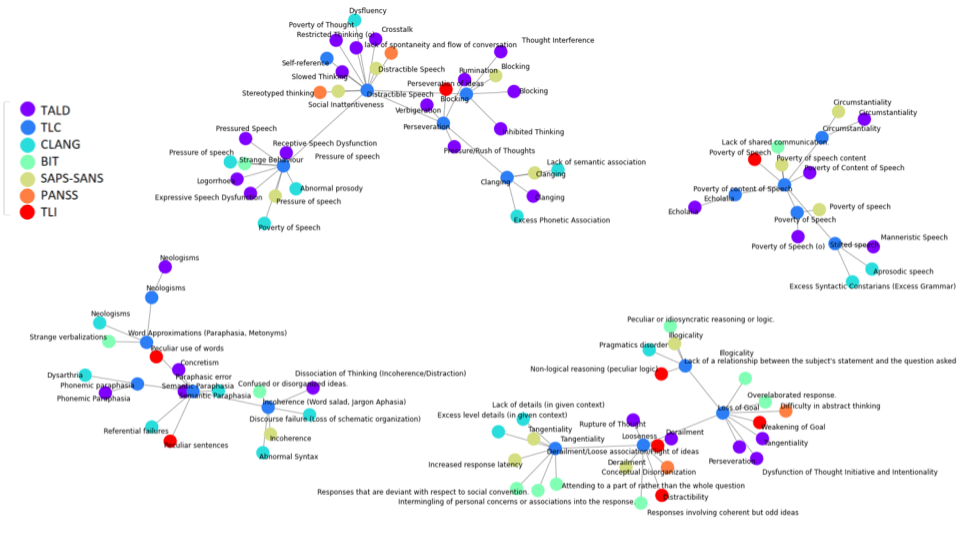
**Supplemental figure 1 - Semantic network of all items to TLC backbone.** Same network as figure 3, but here the length of edges represents semantic similarity between nodes.

**Supplemental table 1** - included Formal thought rating scale items.

| label | Name | Content |
| --- | --- | --- |
| TALD-1 | Circumstantiality | Thinking is circuitous; minor matters cannot be separated from essential matters. The main point gets lost in the description of details, without losing the intentional goal completely (long-winded speech). Insufficient capacity to process abstract information might be one of several causes for Circumstantiality, as well as the inability to omit minor matters even when the patient knows them to be inessential. |
| TALD-2 | Derailment | A pattern of spontaneous speech in which ideas slip "off the track" onto other thoughts which are clearly but obliquely related. Things may be said in juxtaposition which lack a meaningful relationship, or the patient may shift idiosyncratically from one frame of reference to another. At times there may be vague connections between the ideas. The objective characteristic of Derailment should be coded as if the interviewer were talking to the patient for the first time (unaware of potential personal associative connections between the thoughts). One manifestation of this disorder is a slow steady slippage, with no single Derailment being particularly severe, so that the speaker gets farther and farther off the track with each Derailment without any awareness that his reply no longer has any connection to the question being asked. |
| TALD-3 | Tangentiality | Ideas do not follow a straight path. Within longer speech passages, content slowly drifts away from where it originally started. The patient does not return to the initial topic. |
| TALD-4 | Dissociation of Thinking (Incoherence/Distraction) | The content of a phrase, sentence or thought has no reference to what has been said before. In contrast to Derailment (Item 2) where associative bridges are still recognizable, Dissociation of Thinking refers to the state in which words, sentences and thoughts have no relation to each other. In less severe occurrences, single sentences may still make sense; however, coherence between sentences is absent. In the severest occurrences, coherence within a sentence or even within individual words is absent (scattered speech). |
| TALD-5 | Crosstalk | The response of the patient misses the point at hand, although he has understood the question. The evaluation of this item does not depend on whether the answer to the question is wrong or not (like a wrong answer in an examination), but that the patient is talking past the question. If the interviewer has any kind of suspicion with regard to the presence of Crosstalk, it must be verified that the patient has understood the question correctly. Therefore, the patient should be asked to repeat the question. Intentional ignoring of the question ("beating around the bush") should not be considered. |
| TALD-6 | Perseveration | Adherence to previously mentioned ideas and topics that no longer fit the current context. |
| TALD-7 | Verbigeration | Unnecessary repetition of a single word. |
| TALD-8 | Rupture of Thought | Objectively observed sudden interruption of a previously fluid line of thought. The phenomenon may occur in the middle of a sentence and for no apparent reason. |
| TALD-9 | Pressured Speech | The speed of speech production is increased. |
| TALD-10 | Logorrhoea | An excessively strong urge to speak. Logorrhoeic speech itself may be coherent and logical. Accelerated speech production need not be present. Communication with the patient is hindered. The patient is either not able to recognize when he is being interrupted or simply ignores such interruptions. |
| TALD-11 | Manneristic Speech | For the observer, speech (word selection, sentence structure, articulation or prosody) seems affected and ornate, eccentric, unnatural, pompous, overblown, fancy, stylised or flowery. |
| TALD-12 | Semantic Paraphasia | Substitution of an inappropriate word (the word is semantically related to the appropriate word). The speaker may or may not recognize his error and attempt to correct it. |
| TALD-13 | Phonemic Paraphasia | Mispronunciation (with regard to phonetic articulation) of a word. Milder forms may occur as "slips of the tongue" in everyday speech. The speaker usually recognizes his error and may attempt to correct it. |
| TALD-14 | Neologisms | New word formations, which do not correspond to lexical conventions. Most Neologisms are not directly intelligible. In extreme cases a new artificial language can be formed or used by the patient. Expressions or slang words used in particular groups or subcultures (e.g. youth culture, the drug scene) are not to be classified as Neologisms. Insufficient language abilities (e.g. in a non-native speaker) should be excluded as well. |
| TALD-15 | Clanging | A pattern of speech in which sounds, rather than meaningful relationships, appear to govern word choice, so that the intelligibility of the speech is impaired and redundant words are introduced. In addition to rhyming relationships, this pattern of speech may also include punning associations, so that a word similar in sound (polysemy/homophony) brings in a new thought. |
| TALD-16 | Echolalia | Senseless repetitions of words and sentences with no regard to their meanings and semantic functions. The patient echoes the words or sentences of the interviewer. |
| TALD-17 | Poverty of Content of Speech | Although replies are long enough that speech is adequate in amount, it conveys little information. Language tends to be vague, often overly abstract or overly concrete, repetitive, and stereotyped. The interviewer may recognize this finding by observing that the patient has spoken at some length but has not given adequate information to answer the question. Alternatively, the patient may provide enough information, but require many words to do so, so that a lengthy reply can be summarized in a sentence or two. |
| TALD-18 | Restricted Thinking (o) | Restriction in the range of content, adherence to one topic or a few topics, or fixation on a few key ideas. During the conversation, the patient experiences difficulties in switching from one topic to another, or constantly returns to the initial topic. For a successful exploration, it is necessary that the examiner offers the patient a variety of topics. This is important since the topic of illness always forms part of a psychiatric exploration, but this should not automatically be treated as resulting from Restricted Thinking. When exploring the topic of illness, it is only possible to refer to Restricted Thinking when the patient is fixed on single aspects of his illness, and when he is not able to detach from these aspects despite being offered other topics of discussion (e.g. a depressive patient who is preoccupied with his indigestion). |
| TALD-19 | Slowed Thinking | From the observer's perspective, the patients' thought process seems to be slowed down (objective). As a result of this sluggish thinking process, the conversation is languid and torpid. |
| TALD-20 | Poverty of Speech (o) | Restriction in the amount of spontaneous speech, so that answers to given questions tend to be brief, concrete and unelaborated. Unprompted additional information is rarely provided. Replies may be monosyllabic, and some questions may be left unanswered altogether. When confronted with this speech pattern, the interviewer may find himself frequently prompting the patient in order to encourage elaboration of replies. To elicit this finding, the examiner must allow the patient adequate time to answer and to elaborate his answer. |
| TALD-21 | Concretism | Concretism refers to difficulty in the comprehension of abstract (figurative) sentences or phrases (e.g. the understanding/interpretation of proverbs, metaphors, jokes). The patient adheres to the concrete meaning of the words/utterances. |
| TALD-22 | Blocking | Perceived and reported blocking of an ongoing line of thought, also known as "losing one's train of thought". Blocking is subjectively noticed by the patient. Included is the phenomenon of "Fading", which refers to a slow dimming away of a thought, as opposed to a sudden termination (in the sense of "Rupture of Thought," Item 1). Fading can also occur in a fluctuating manner, which means that the thought becomes weaker at first, then becomes clearer again, then fades away once more. In contrast to a loss of thought, Fading can only be assessed if the patient himself reports this phenomenon. Blocking and Fading may occur either with or without Thought Interference (Item 22, infiltration of a new thought). |
| TALD-23 | Rumination | The patient is constantly occupied with mostly unpleasant topics. These thoughts center around the same topics without leading to any conclusion. For the patient, it is hard to interrupt these negative thought processes. Rumination is experienced as unpleasant and in some cases even torturous |
| TALD-24 | Poverty of Thought | The patient has the sense that his thinking is unimaginative and restricted to just a few themes. This may or may not be accompanied by unpleasant feelings. |
| TALD-25 | Inhibited Thinking | The process of thinking is experienced by the patient as being slowed down, braked or inhibited, as if he is thinking against an internal resistance. The patient is not able to overcome this inhibition of his thoughts. Inhibited Thinking can extend to the point that the patient has the subjective experience of not being able to think at all anymore. |
| TALD-26 | Receptive Speech Dysfunction | The meanings of words, word sequences or sentences (for example, in conversations, movies and radio programs) can only be grasped or understood incompletely, with effort, or not at all. In some instances, the phenomenon may only occur after some time of mental strain. However, in other instances, the rate of verbal processing may already be reduced from the start. As a result of the disordered receptive speech abilities, the patient may have problems with interpersonal communication |
| TALD-27 | Expressive Speech Dysfunction | The patient notices that it is difficult for him to find the right words. While the patient is speaking, he notices that word choice, linguistic precision and word fluency are affected. Appropriate words are not quickly accessible or are completely unavailable. In some cases only imprecise and unclear formulations come to mind. In severe manifestations of this phenomenon, self-experienced Crosstalk phenomena can occur, which may result in tactless or inappropriate utterances. Some patients may try to compensate for the disorder by repeating empty phrases and verbiage, or they may even avoid conversation altogether. |
| TALD-28 | Dysfunction of Thought Initiative and Intentionality | The patient's subjective experiences a lack of thought initiative, "thought energy" and intentionality; he is aware of the dysfunction. The impairment in initiating or structuring the thought process may result (due to the lack of an overarching concept) in the inability to perform activities of daily living (e.g. making coffee). |
| TALD-29 | Thought Interference | Interfering thoughts or ideas that do not belong to the current line of thought. Thought Interference may or may not be triggered by or linked to external stimuli. The interfering thoughts are more or less neutral in terms of their affective weighting. |
| TALD-30 | Pressure/Rush of Thoughts | Numerous thoughts with varied content jump into or impose on the patient's mind, alternating rapidly. The patient is able to neither control nor suppress these appearing and disappearing thoughts. |
| SAPS-SANS-9 | Poverty of speech | Restriction in the amount of spontaneous speech, so that replies to questions tend to be brief, concrete, and unelaborated. Unprompted additional information is rarely provided. Replies may be monosyllabic, and some questions may be left unanswered altogether. When confronted with this speech pattern, the interviewer may find himself frequently prompting the subject in order to encourage elaboration of replies. To elicit this finding, the examiner must allow the subject adequate time to answer and to elaborate his answer |
| SAPS-SANS-10 | Poverty of speech content | Although replies are long enough so that speech is adequate in amount, it conveys little information. Language tends to be vague, often over-abstract or over-concrete, repetitive, and stereotyped. The interviewer may recognize this finding by observing that the subject has spoken at some length but has not given adequate information to answer the question. Alternatively, the subject may provide enough information, but require many words to do so, so that a lengthy reply can be summarized in a sentence or two. Sometimes the interviewer may characterize the speech as "empty philosophizing." |
| SAPS-SANS-11 | Blocking | Interruption of a train of speech before a thought or idea has been completed. After a period of silence which may last from a few seconds to minutes, the person indicates that she/he cannot recall what he had been saying or meant to say. Blocking should only be judged to be present if a person voluntarily describes losing his thought or if, upon questioning by the interviewer, the person indicates that that was the reason for pausing. |
| SAPS-SANS-12 | Increased response latency | The subject takes a longer time to reply to questions than is usually considered normal. He may seem "distant" and sometimes the examiner may wonder if he has even heard the question. Prompting usually indicates that the subject is aware of the question, but has been having difficulty in formulating his thoughts in order to make an appropriate reply. |
| SAPS-SANS-23 | Social Inattentiveness | While involved in social situations or activities, the subject appears inattentive. He looks away during conversations, does not pick up the topic during a discussion, or appears uninvolved or unengaged. He may abruptly terminate a discussion or a task without any apparent reason. He may seem "spacy" or "out of it". He may seem to have poor concentration when playing games, reading, or watching TV. |
| SAPS-SANS-26 | Derailment | A pattern of spontaneous speech in which the ideas slip off one track onto another which is clearly but obliquely related, or onto one which is completely unrelated. Things may be said in juxtaposition which lack a meaningful relationship, or the subject may shift idiosyncratically from one frame of reference to another. At times there may be a vague connection between the ideas, and at others none will be apparent. This pattern of speech is often characterized as sounding "disjointed." Perhaps the commonest manifestation of this disorder is a slow, steady slippage, with no single derailment being particularly severe, so that the speaker gets farther and farther off the track with each derailment without showing any awareness that his reply no longer has any connection with the question which was asked. This abnormality is often characterized by lack of cohesion between clauses and sentences and by unclear pronoun references |
| SAPS-SANS-27 | Tangentiality | Replying to a question in an oblique, tangential or even irrelevant manner. The reply may be related to the question in some distant way. Or the reply may be unrelated and seem totally irrelevant. In the past tangentiality has sometimes been used as roughly equivalent to loose associations or derailment. The concept of tangentiality has been partially redefined so that it refers only to answers to questions and not to transitions in spontaneous speech. |
| SAPS-SANS-28 | Incoherence | A pattern of speech which is essentially incomprehensible at times. Incoherence is often accompanied by derailment. It differs from derailment in that in incoherence the abnormality occurs within the level of the sentence or clause, which contains words or phrases that are joined incoherently. The abnormality in derailment involves unclear or confusing connections between larger units, such as sentences or clauses. This type of language disorder is relatively rare. When it occurs, it tends to be severe or extreme, and mild forms are quite uncommon. It may sound quite similar to Wernicke's aphasia or jargon aphasia, and in these cases the disorder should only be called incoherence when history and laboratory data exclude the possibility of a past stroke, and formal testing for aphasia is negative. |
| SAPS-SANS-29 | Illogicality | A pattern of speech in which conclusions are reached which do not follow logically. This may take the form of non-sequiturs (= it does not follow), in which the subject makes a logical inference between two clauses which is unwarranted or illogical. It may take the form of faulty inductive inferences. It may also take the form of reaching conclusions based on faulty premises without any actual delusional thinking |
| SAPS-SANS-30 | Circumstantiality | A pattern of speech which is very indirect and delayed in reaching its goal idea. In the process of explaining something, the speaker brings in many tedious details and sometimes makes parenthetical remarks. Circumstantial replies or statements may last for many minutes if the speaker is not interrupted and urged to get to the point. Interviewers will often recognize circumstantiality on the basis of needing to interrupt the speaker in order to complete the process of history- taking within an allotted time. When not called circumstantial, these people are often referred to as "long-winded." |
| SAPS-SANS-31 | Pressure of speech | An increase in the amount of spontaneous speech as compared to what is considered ordinary or socially customary. The subject talks rapidly and is difficult to interrupt. Some sentences may be left uncompleted because of eagerness to get on to a new idea. Simple questions which could be answered in only a few words or sentences are answered at great length so that the answer takes minutes rather than seconds and indeed may not stop at all if the speaker is not interrupted. Even when interrupted, the speaker often continues to talk. Speech tends to be loud and emphatic. Sometimes speakers with severe pressure will talk without any social stimulation and talk even though no one is listening. When subjects are receiving phenothiazines or lithium, their speech is often slowed down by medication, and then it can be judged only on the basis of amount, volume, and social appropriateness. If a quantitative measure is applied to the rate of speech, then a rate greater than 150 words per minute is usually considered rapid or pressured. This disorder may be accompanied by derailment, tangentiality, or incoherence, but it is distinct from them. |
| SAPS-SANS-32 | Distractible Speech | During the course of a discussion or interview, the subject stops talking in the middle of a sentence or idea and changes the subject in response to a nearby stimulus, such as an object on a desk, the interviewer's clothing or appearance, etc. |
| SAPS-SANS-33 | Clanging | A pattern of speech in which sounds rather than meaningful relationships appear to govern word choice, so that the intelligibility of the speech is impaired and redundant words are introduced. In addition to rhyming relationships, this pattern of speech may also include punning associations, so that a word similar in sound brings in a new thought. |
| PANSS-P2 | Conceptual Disorganization | Disorganized process of thinking characterized by disruption of goal-directed sequencing, e.g., circumstantiality, tangentiality, loose associations non sequiturs, gross illogicality, or thought block. Basis for rating: cognitive-verbal processes observed during the course of interview. |
| PANSS-N5 | Difficulty in abstract thinking | Impairment in the use of the abstract-symbolic mode of thinking, as evidenced by difficulty in classification, forming generalizations, and proceeding beyond concrete or egocentric thinking in problem solving tasks. Basis for rating: responses to questions on similarities and proverb interpretation, and use of concrete vs. abstract mode during the course of the interview. |
| PANSS-N6 | lack of spontaneity and flow of conversation | Reduction in the normal flow of communication associated with apathy, avolition, defensiveness, or cognitive deficit. This is manifested by diminished fluidity and productivity of the verbal-interactional process. Basis for rating: cognitive-verbal processes observed during the course of interview. |
| PANSS-N7 | Stereotyped thinking | Decreased fluidity, spontaneity, and flexibility of thinking, as evidenced in rigid, repetitious, or barren thought content. Basis for rating: cognitive verbal processes observed during the interview |
| TLC-1 | Poverty of Speech | Restriction in the amount of spontaneous speech, so that replies to questions tend to be brief, concrete, and unelaborated. Unprompted additional information is rarely provided. Replies may be monosyllabic, and some questions may be left unanswered altogether. When confronted with this speech pattern, the interviewer may find himself frequently prompting the patient in order to encourage elaboration of replies. To elicit this finding, the examiner must allow the patient adequate time to answer and to elaborate his answer. |
| TLC-2 | Poverty of content of Speech | Although replies are long enough so that speech is adequate in amount, it conveys little information. Language tends to be vague, often overabstract or overconcrete, repetitive, and stereotyped. The interviewer may recognize this finding by observing that the patient has spoken at some length but has not given adequate information to answer the question. Alternatively, the patient may provide enough information, but require many words to do so, so that a lengthy reply can be summarized in a sentence or two. Sometimes the interviewer may characterize the speech as "empty philosophizing." |
| TLC-3 | Pressure of speech | An increase in the amount of spontaneous speech as compared to what is considered ordinary or socially customary. The patient talks rapidly and is difficult to interrupt. Some sentences may be left uncompleted because of eagerness to get on to a new idea. Simple questions which could be answered in only a few words or sentences are answered at great length so that the answer takes minutes rather than seconds and indeed may not stop at all if the speaker is not interrupted. Even when interrupted, the speaker often continues to talk. Speech tends to be loud and emphatic. Sometimes speakers with severe pressure will talk without any social stimulation and talk even though no one is listening. When patients are receiving phenothiazines or lithium, the speech is often slowed down by medication, and then it can be judged only on the basis of amount, volume, and social appropriateness. If a quantitative measure is applied to the rate of speech, then a rate greater than 150 words/minute is usually considered rapid or pressured. This disorder may be accompanied by derailment, tangentiality, or incoherence, but it is distinct from them. |
| TLC-4 | Distractible Speech | During the course of a discussion or interview, the patient stops talking in the middle of a sentence or idea and changes the subject in response to a nearby stimulus, such as an object on a desk, the interviewer's clothing or appearance, etc. |
| TLC-5 | Tangentiality | Replying to a question in an oblique, tangential, or even irrelevant manner. The reply may be related to the question in some distant way. Or the reply may be unrelated and seem totally irrelevant. In the past tangentiality has been used as roughly equivalent to loose associations or derailment. The concept of tangentiality has been partially redefined so that it refers only to replies to questions and not to transitions in spontaneous speech |
| TLC-6 | Derailment/Loose association/Flight of ideas | A pattern of spontaneous speech in which the ideas slip off the track onto another one which is clearly but obliquely related, or onto one which is completely unrelated. Things may be said in juxtaposition which lack a meaningful relationship, or the patient may shift idiosyncratically from one frame of reference to another. At times there may be a vague connection between the ideas, and at others none will be apparent. This pattern of speech is often characterized as sounding "disjointed. ' Perhaps the commonest manifestation of this disorder is a slow, steady slippage, with no single derailment being particularly severe, so that the speaker gets farther and farther off the track with each derailment without showing any awareness that his reply no longer has any connection with the question which was asked This abnormality is often characterized by lack of cohesion between clauses and sentences and by unclear pronoun referents. Although less severe derailments (i.e., those in which the relationship between juxtaposed ideas is oblique) have sometimes been referred to in the past as tangentiality or as flight of ideas when in the context of mania, such distinctions are not recommended because they tend to be unreliable. Flight of ideas is a derailment which occurs rapidly in the context of pressured speech. Tangentiality has been defined herein as a different phenomenon in that it occurs as the immediate response to a question. |
| TLC-7 | Incoherence (Word salad, Jargon Aphasia) | A pattern of speech which is essentially incomprehensible at times. The incoherence is due to several different mechanisms, which may sometimes all occur simultaneously. Sometimes portions of coherent sentences may be observed in the midst of a sentence which is incoherent as a whole. Sometimes the disturbance appears to be at a semantic level, so that words are substituted in a phrase or sentence so that the meaning seems to be distorted or destroyed; the word choice may seem totally random or may appear to have some oblique connection with the context. Sometimes "cementing words" (coordinating and subordinating conjunctions such as "and," "although"; adjectival pronouns such as "the," "a," and "an") are deleted. Incoherence is often accompanied by derailment. It differs from derailment in that the abnormality in incoherence occurs within the level of the sentence or clause, which contains words or phrases that are joined incoherently. The abnormality in derailment involves unclear or confusing connections between larger units, such as sentences or clauses. This type of language disorder is relatively rare. When it occurs, it tends to be severe or extreme, and mild forms are quite uncommon. It may sound quite similar to a Wernicke's aphasia or jargon aphasia, and in these cases the disorder should only be called incoherence (thereby implying a psychiatric disorder as opposed to a neurological disorder) when history and laboratory data exclude the possibility of a known organic etiology and formal testing for aphasia is negative. |
| TLC-8 | Illogicality | A pattern of speech in which conclusions are reached which do not follow logically. This may take the form of non sequiturs (= it does not follow), in which the patient makes a logical inference between two clauses which is unwarranted or illogical. It may take the form of faulty inductive inferences. It may also take the form of reaching conclusions based on faulty premises without any actual delusional thinking. |
| TLC-9 | Clanging | A pattern of speech in which sounds rather than meaningful relationships appear to govern word choice, so that the intelligibility of the speech is impaired and redundant words are introduced. In addition to rhyming relationships, this pattern of speech may also include punning associations, so that a word similar in sound brings in a new thought. |
| TLC-10 | Neologisms | New word formations. A neologism is defined here as a completely new word or phrase whose derivation cannot be understood. Sometimes the term "neologism" has also been used to mean a word which has been incorrectly built up but with origins which are understandable as due to a misuse of the accepted methods of word formation. For purposes of clarity, these should be referred to as word approximations (q.v.). Neologisms are quite uncommon. |
| TLC-11 | Word Approximations (Paraphasia, Metonyms) | Old words which are used in a new and unconventional way, or new words which are developed by conventional rules of word formation. Often the meaning will be evident even though the usage seems peculiar or bizarre (i.e., a ballpoint pen referred to as "paperskate," etc.). Sometimes the word approximations may be based on the use of stock words, so that the patient uses one or several words repeatedly in ways that give them a new meaning (i.e., a watch may be called a "time vessel," the stomach a "food vessel," a television set a "news vessel," etc.). |
| TLC-12 | Circumstantiality | A pattern of speech which is very indirect and delayed in reaching its goal idea. In the process of explaining something, the speaker brings in many tedious details and sometimes makes parenthetical remarks. Circumstantial replies or statements may last for many minutes if the speaker is not interrupted and urged to get to the point. Interviewers will often recognize circumstantiality on the basis of needing to interrupt the speaker in order to complete the process of history-taking within an allotted time. When not called circumstantial, these people are often referred to as "long-winded." |
| TLC-13 | Loss of Goal | Failure to follow a chain of thought through to its natural conclusion. This is usually manifested in speech which begins with a particular subject, wanders away from the subject, and never returns to it. The patient may or may not be aware that he has lost his goal. This often occurs in association with derailment. |
| TLC-14 | Perseveration | Persistent repetition of words, ideas, or subjects so that, once a patient begins a particular subject or uses a particular word, he continually returns to it in the process of speaking. |
| TLC-15 | Echolalia | A pattern of speech in which the patient echoes the words or phrases of the interviewer. Typical echolalia tends to be repetitive and persistent. The echo is often uttered with a mocking, mumbling, or staccato intonation. Echolalia is relatively uncommon in adults, but more frequent in children. |
| TLC-16 | Blocking | Interruption of a train of speech before a thought or idea has been completed. After a period of silence, which may last from a few seconds to minutes, the person indicates that he cannot recall what he had been saying or meant to say. Blocking should only be judged to be present either if a person voluntarily describes losing his thought or if upon questioning by the interviewer, the person indicates that that was his reason for pausing. |
| TLC-17 | Stilted speech | Speech which has an excessively stilted or formal quality. It may seem rather quaint or outdated, or it may appear pompous, distant, or overly polite. The stilted quality is usually achieved through the use of particular word choices (multisyllabic when monosyllabic alternatives are available and equally appropriate), extremely polite phraseology, or stiff and formal syntax. |
| TLC-18 | Self-reference | A disorder in which the patient repeatedly refers the subject under discussion back to himself when someone else is talking and also refers apparently neutral subjects to himself when he himself is talking. This finding usually cannot be evaluated on the basis of a psychiatric interview, since the subject is then asked to talk about himself. It may be observed during the tests of the sensorium or informal conversation about neutral subjects and should be rated only in that context. |
| TLC-19 | Phonemic paraphasia | Recognizable mispronunciation of a word because sounds or syllables have slipped out of sequence. Severe forms occur in aphasia, but milder forms may occur as "slips of the tongue" in everyday speech. The speaker usually recognizes his error and may attempt to correct it. |
| TLC-20 | Semantic Paraphasia | Substitution of an inappropriate word when trying to say something specific. The speaker may or may not recognize his error and attempt to correct it. This typically occurs in both Broca's and Wernicke's aphasia. It may be difficult to distinguish from incoherence since incoherence may also be due to semantic substitutions which distort or obscure meaning; when this differential decision must be made, it is suggested that formal testing for aphasia be completed; if the testing is positive, then the semantic substitutions may be considered due to semantic paraphasia, and if negative to incoherence. |
| TLI-1 | Poverty of Speech | This item refers to a decrease in the amount of speech. Responses to the picture and replies to questions are brief and lack elaboration. (If there is a lack of adjectives, adverbs or qualitative information, weakening of goal 0.25 might be scored in addition to the score for poverty of speech) |
| TLI-2 | Weakening of Goal | Weakening of goal reflects a lack of drive in thinking and is manifest in lack of normal elaboration of ideas, use of uninformative generalizations and empty speech that conveys little information. It differs from instability of goal (manifest in phenomena such as derailment) in which the problem is one of focusing on one of several competing goals. In contrast, weakening of goal is associated with a diminished amount of thought. Thus, the cardinal features of the speech of patient with weakening of goal are lack of the relevant detail and / or excessive use of vacuous phrases that convey little information. In normal speech, phrases bearing little information are interspersed within a stream of informative phrases, apparently as a means of maintaining contact between speaker and listener while thoughts are being marshalled; the use of such empty phrases reflects abnormality only when they occupy a large proportion of the total speech. Weakening of goal might be accompanied by poverty of speech (diminished amount of speech), but not necessarily so. The essential issue is lack of information in proportion to the number of words spoken. |
| TLI-3 | Perseveration of ideas | Unwarranted repetition of ideas or themes. Increased weighting is given to instances in which the repetition occurs independently of the stimulus provided by the picture. |
| TLI-4 | Looseness | The connection between ideas is tenuous or absent, or extraneous ideas intrude into the train of thought. Looseness can be manifest during a spontaneous train of thought (designated derailment by Andreasen), or by virtue of irrelevant or tangential replies to questions (designated tangentiality by Andreasen). |
| TLI-5 | Peculiar use of words | Use of words that are unusual, or use of invented words. If the word is used more than once in the response to a single picture, rate only the first use, but consider the possibility of scoring perseveration of ideas when the inappropriate word is used repeatedly |
| TLI-6 | Peculiar sentences | The form of the sentences is unusual. Greater weight is given to peculiarity of sentence construction which makes it difficult to discern the meaning. Sentences with grammatical inaccuracies which can be attributed to poor education, should not be rated as peculiar. |
| TLI-7 | Non-logical reasoning (peculiar logic) | Reaching conclusions based on inadequate evidence or faulty logic. The judgment that an utterance reflects peculiar logic should be made after the enquiry phase. It is essential that the examiner should note all unusual ideas expressed during the free response phase, and make a subsequent enquiry into the basis for the these ideas. Allow reasonable speculation. If an inappropriate utterance is based only on perseveration, it is not rated as peculiar logic. However, if a subject gives an irrational explanation for an instance of perseveration, occurrences of both perseveration and peculiar logic should be recorded |
| TLI-8 | Distractibility | Intrusion of extraneous ideas arising from an external stimulus |
| CLANG-1 | Excess Phonetic Association | Abnormal association based on phonetic similarity (punning and clang associations) |
| CLANG-2 | Abnormal Syntax | Violations of ordinary rules of grammar leading to incomprehensible speech |
| CLANG-3 | Excess Syntactic Constarians (Excess Grammar) | Excessive application of rigid grammatical structure to speech output, producing language that is "formal" and lack of flexibility of ordinary spoken language |
| CLANG-4 | Lack of semantic association | Lack of normal semantic relationship between ideas expressed successively |
| CLANG-5 | Referential failures | Unclear links (anaphoric) which leave excessive ambiguity as to which expressions refers back (or forth) to which items in preceding and subsequent speech |
| CLANG-6 | Discourse failure (Loss of schematic organization) | Lack of the normal organization in which larger speech units (e.g. one or two sentences or above) progresses from one context to the next in a gradual and prepared manner. this abnormality results in speech that loss overall goals and direction although relationship at the level of smaller units (words and phrases within one or two sentences) may be considered normal |
| CLANG-7 | Excess level details (in given context) | Details given grossly in excess of that required in the given context. |
| CLANG-8 | Lack of details (in given context) | details given (though judged to be probably appropriate in meaning) grossly inadequate to context. |
| CLANG-9 | Aprosodic speech | Flat monotonous speech without appropriate inflexion and emotional quality |
| CLANG-10 | Abnormal prosody | Bizarre quality of voice e.g. high-pitch, mechanical etc. |
| CLANG-11 | Pragmatics disorder | Speech content reflects defective knowledge of the world (judged to be independent of delusional ideas i.e. of personal significance etc.) |
| CLANG-12 | Dysfluency | Stuttering, false starts, hesitations |
| CLANG-13 | Dysarthria | articulation difficulties |
| CLANG-14 | Poverty of Speech | reduced overall speech output |
| CLANG-15 | Pressure of speech | increased speed of speech production, as if speech is paced by a rapid internal production process |
| CLANG-16 | Neologisms | Construction of idiosyncratic new words for personal use. |
| CLANG-17 | Paraphasic error | substitution of word by words with similar meaning (but inappropriate and less precise). |
| BIT-1 | Strange verbalizations | Single words used in an unusual or peculiar manner (which are, in the rater's best judgment, not attributable to intellectual or cultural deficits). Mild or moderate cognitive slippage in sentence structure, the expression of ideas, or the construction of new words (the new word is close in form to the correct word). Neologisms (a new word with private meaning). Real neologisms (involving a private meaning) are very unusual, and are scored "3" to reflect their very severe idiosyncratic quality. Artificial, pedantic, or stilted language, inappropriate to the level of discourse in the testing situation. |
| BIT-2 | Lack of shared communication. | Responses that are not explicitly stated. Small gaps in communication, in which words are not explained or referents are unclear. Larger gaps in communication, in which phrases are not explained. Elements of private language may be apparent with unshared or unexplained concepts or ideas. Disorganized or poor linkage between consecutive words, phrases, or sentences within the response. |
| BIT-3 | Responses involving coherent but odd ideas | Responses involving coherent but odd ideas |
| BIT-4 | Responses that are deviant with respect to social convention. | Responses that are deviant with respect to social convention. |
| BIT-5 | Peculiar or idiosyncratic reasoning or logic. | Responses that are incorrect and illogical in terms of common knowledge about people, events, or the environment. Responses violating a logical paradigm, such as predicate logic. Self-contradictory responses or responses with confused logic. Responses with peculiar, autistic logic. |
| BIT-6 | Confused or disorganized ideas. | Combinations of words put together in a manner that only dimly makes sense. Grammatically correct sentences that do not hold a logical thought. |
| BIT-7 | Overelaborated response. | Irrelevant wandering within a partially correct or correct answer. Elaboration that is far too extensive, to the point where the original question is almost lost from sight. |
| BIT-8 | Intermingling of personal concerns or associations into the response. | Intermingling of personal concerns or associations into the response. |
| BIT-9 | Attending to a part of rather than the whole question | associations or interpretations of a word or phrase that suggest the subject's response is not based on the question as a conceptual whole, and also make the response appear strange or idiosyncratic. |
| BIT-10 | Lack of a relationship between the subject's statement and the question asked | Lack of a relationship between the subject's statement and the question asked‚ almost as if a different question is being asked. |
| BIT-11 | Strange Behaviour | Strange behavior‚ including physical and affective behavior. |
